# Asteltoxins with Antiviral Activities from the Marine Sponge-Derived Fungus *Aspergillus* sp. SCSIO XWS02F40

**DOI:** 10.3390/molecules21010034

**Published:** 2015-12-26

**Authors:** Yong-Qi Tian, Xiu-Ping Lin, Zhen Wang, Xue-Feng Zhou, Xiao-Chu Qin, Kumaravel Kaliyaperumal, Tian-Yu Zhang, Zheng-Chao Tu, Yonghong Liu

**Affiliations:** 1 CAS Key Laboratory of Tropical Marine Bio-resources and Ecology/Guangdong Key Laboratory of Marine Materia Medica/RNAM Center for Marine Microbiology, South China Sea Institute of Oceanology, Chinese Academy of Sciences, Guangzhou 510301, China; tian.yongqi@163.com (Y.-Q.T.); xiupinglin@hotmail.com (X.-P.L.); zhou-xuefeng@hotmail.com (X.-F.Z.); kumar_bio06@yahoo.co.in (K.K.); 2 University of Chinese Academy of Sciences, Beijing 100049, China; 3 Laboratory of Molecular Engineering and Laboratory of Natural Product Synthesis, Guangzhou Institutes of Biomedicine and Health, Chinese Academy of Sciences, Guangzhou 510530, China; wang_zhen2013@gibh.ac.cn (Z.W.); qin_xiaochu@gibh.ac.cn (X.-C.Q.); tu_zhengchao@gibh.ac.cn (Z.-C.T.); 4 Laboratory of State Key Laboratory of Respiratory Disease, Guangzhou Institutes of Biomedicine and Health, Chinese Academy of Sciences, Guangzhou 510530, China; zhang_tianyu@gibh.ac.cn

**Keywords:** sponge-derived fungus, asteltoxins, antiviral (H1N1 and H3N2) activity

## Abstract

Two new asteltoxins named asteltoxin E (**2**) and F (**3**), and a new chromone (**4**), together with four known compounds were isolated from a marine sponge–derived fungus, *Aspergillus* sp. SCSIO XWS02F40. The structures of the compounds (**1**–**7**) were determined by the extensive 1D- and 2D-NMR spectra, and HRESIMS spectrometry. All the compounds were tested for their antiviral (H1N1 and H3N2) activity. Compounds **2** and **3** showed significant activity against H3N2 with the prominent IC_50_ values of 6.2 ± 0.08 and 8.9 ± 0.3 μM, respectively. In addition, compound **2** also exhibited inhibitory activity against H1N1 with an IC_50_ value of 3.5 ± 1.3 μM.

## 1. Introduction

In 2015, viral pathogenesis became an intriguing hot topic among human society. This is exemplified by the recent Ebola virus outbreak in Western Africa and Middle East Respiratory Syndrome (MERS) in South Korea [1]. Meanwhile, influenza viruses are still a great threat to human health [2,3]. So far, only two classes of antiviral drugs, which include amantadine and the neuraminidase inhibitors, are currently used as anti-influenza therapeutic drugs, but both of them have some adverse side effects in humans as well as the resistance of the virus towards this drug [4,5]. Hence, a concerted effort is aimed at discovering new antiviral agents to treat and eradicate these infectious agents [6,7].

**Figure 1:**
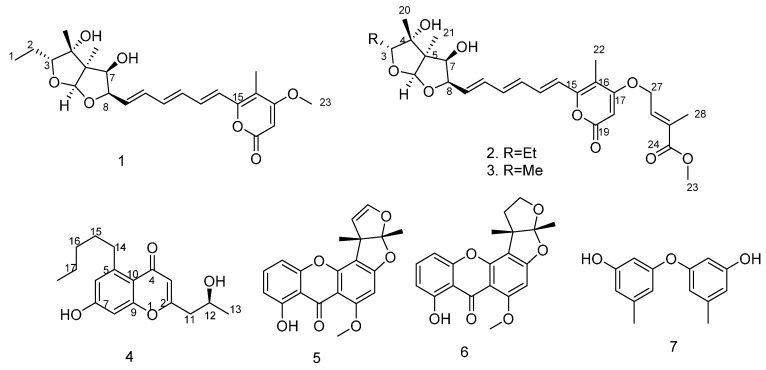
Structure of compounds **1**–**7**.

Marine sponge–derived fungus tends to produce structurally unique and biologically active natural products which have been documented in recent years; however, only a handful of reports have described new metabolites which have antiviral activities [7,8]. To further the scope of this particular research theme, a fungus, *Aspergillus* sp. SCSIO XWS02F40, attracted our attention because its crude EtOAc extract exhibited potential antiviral activity. Further isolation yielded three new compounds: asteltoxin E (**2**), asteltoxin F (**3**) and 7-hydroxy-2-(2-hydroxypropyl)-5-pentylchro mone (**4**), together with four known compounds: asteltoxin (**1**) [9], sterigmatocystin (**5**) [10,11], dihydrosterigmatocystin (**6**) [11,12], diorcinol (**7**) [13,14] (Figure 1). The antiviral (H1N1 and H3N2) activities of these compounds were individually evaluated. Herein, we described the isolation, structural elucidation, and bioactivity screening of these metabolites **1**–**7** from *Aspergillus* sp. SCSIO XWS02F40.

## 2. Results and Discussion

### 2.1. Structural Elucidation

**Figure 2:**
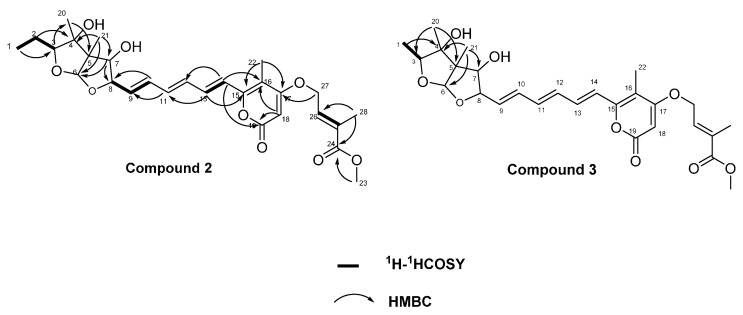
Selected ^1^H-^1^H COSY and HMBC correlations of **2** and **3**.

Compound **2** was obtained as a yellow amorphous solid. The molecular formula of **2** was established as C_28_H_36_O_9_ by the HRESIMS data (*m*/*z* 517.2440 [M + H]^+^), requiring 11 degrees of unsaturations. The UV spectrum showed maxima at 356, 300, 267, 211 nm, indicating the presence of an extended conjugated moiety. The IR spectrum exhibited absorptions for a hydroxy group at 3390 cm^−1^ and an α,β-unsaturated carbonyl group at 1697 cm^−1^ [15]. Close inspection of the ^1^H- and ^13^C-NMR spectra (Table 1, Figures S1 and S2) of **2** using DEPT and ^1^H-^13^C correlation spectroscopy (HSQC) revealed the presence of six methyls (one oxygenated), two methylenes including one oxygen-bearing, 12 methines including three oxygenated methines, one double oxygenated methine (δ_H_ 5.27, δ_C_ 111.9) and eight olefinic methines ,as well as two sp^3^ quaternary carbons (one oxygenated), four sp^2^ quaternary carbons, including two oxygenated carbons (δ_C_ 154.7, 169.5) and two ester carbonyls (δ_C_ 163.7, 167.5). Comparing UV-vis and NMR data of compound **2** with those of asteltoxin (**1**) [9,16,17] revealed a high degree of similarity and a difference in the substituents of C-17. HMBC correlations observed from H_3_-28/C-24, C-25, C-26, H_3_-23/C-24 and H_2_-27/C-24, C-25, C-26 indicated the existence of a methyl crotonate moiety. In addition to this, the HMBC correlation of H_2_-27/C-17 and the chemical shift values of C-17 (δ_C_ 169.5) and C-27 (δ_C_ 65.9) suggested that C-27 was linked with C-17 through an oxygen bridge. Based on these analyses, the planar structure of **2** was determined to be as shown in Figure 2.

**Figure 3:**
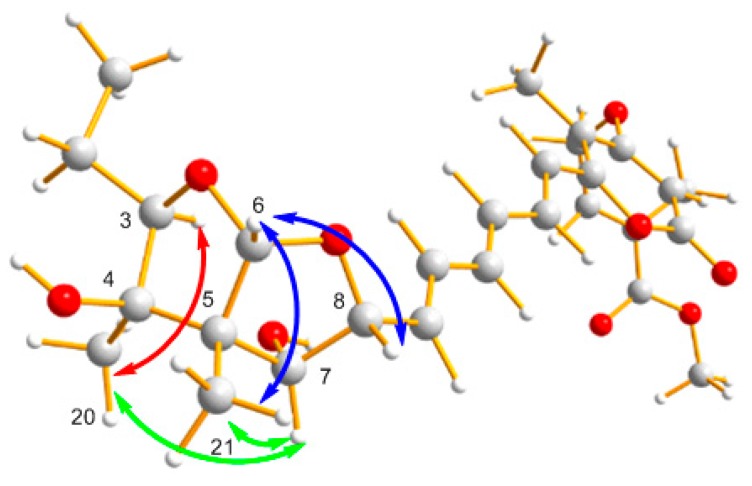
Key NOESY correlations of **1**.

The relative stereochemistry of (**2**) was deduced from NOESY correlations as shown in Figure 3. The double-bond geometry of the C-25/C-26 of substituent was deduced from the NOESY correlations of H_3_-28/H_2_-27 and H-26/H_3_-23, which suggested E-configurations. The NOESY correlations of H-6/H_3_-21 revealed the *cis* fusion of the 2,8-dioxabi cyclo [3.3.0] octane. The NOESY correlations from H-6 to H-8; H-7 to H-8, H_3_-21 suggested that H-6, H-7, H-8 and H_3_-21 are positioned on the same side. The NOESY correlation of H-7/H_3_-20, as well as the lack of NOESY correlation between H_3_-20/H_3_-21, indicated that 4-OH was an α-configuration. The NOESY correlation of H_3_-20/H-3 indicated that the ethyl group at C-3 was in the α-configuration. So, the relative configuration of **2** would be identical with that of an asteltoxin and named asteltoxin E [18].

**Table 1:** The ^1^H- and ^13^C-NMR Data for **1**, **2** and **3** (500/125 MHz, respectively in CDCl_3_, δ ppm, *J* in Hz).

Position	1		2		3	
	δ_c_	δ_H_	δ_c_	δ_H_	δ_c_	δ_H_
1	11.5, CH_3_	1.05, t (7.5)	11.4, CH_3_	1.03, t (7.5)	12.9, CH_3_	1.17, d (6.5)
2	21.8, CH_2_	1.56, m	21.8, CH_2_	1.56, m		
3	89.9, CH	4.31, dd (5.0, 8.0)	89.9, CH	4.31, dd, (5.0, 8.0)	83.8, CH	4.57, q (6.0)
4	81.2, C		81.1, C		81.2, C	
5	62.4 C		62.3, C		62.0, C	
6	111.9, CH	5.29, s	111.9, CH	5.27, s	119.9, CH	5.26, s
7	78.8, CH	3.73, d (2.0)	78.9, CH	3.73, d (2.0)	78.7, CH	3.72, br s
8	83.2, CH	4.74, br.s	83.3, CH	4.70, m	83.1, CH	4.73, br s
9	129.4, CH	5.86, dd (5.0, 15.0)	129.9, CH	5.88, dd (5.5, 15.5)	129.3, CH	5.84, dd (5.0, 15.5)
10	134.2, CH	6.63, dd (11.5, 15.0)	133.9, CH	6.59, dd (11.0, 15.0)	134.2, CH	6.64, dd (11.0, 15.0)
11	136.5, CH	6.49, dd (11.0, 15.0)	136.8, CH	6.46, dd (11.0, 15.0)	136.6, CH	6.49, dd (11.0, 15.0)
12	133.0, CH	6.40, dd (11.0, 15.0)	132.8, CH	6.37, dd (11.0, 15.0)	133.0, CH	6.40, dd (11.0, 15.0)
13	135.5, CH	7.16, dd (11.0, 15.0)	135.8, CH	7.12, dd (11.5, 15.0)	135.8, CH	7.16, dd (11.0, 15.0)
14	120.4, CH	6.40, d (15.0)	120.1, CH	6.36, dd (11.0, 15.0)	120.3, CH	6.37, dd (11.0, 15.0)
15	154.3, C		154.7, C		154.7, C	
16	108.6, C		108.4, C		108.5, C	
17	170.7, C		169.5, C		169.4, C	
18	89.2, CH	5.51, s	89.9, CH	5.45, s	90.1, CH	5.44, s
19	163.9, C		163.7, C		163.6, C	
20	18.1, CH_3_	1.38, s	18.1, CH_3_	1.37, s	17.6, CH_3_	1.34, s
21	16.2, CH_3_	1.18, s	16.2, CH_3_	1.17, s	16.5, CH_3_	1.20, s
22	9.13, CH_3_	1.97, s	9.18, CH_3_	1.97, s	9.23, CH_3_	1.98, s
23	56.4, CH_3_	3.83, s	52.4, CH_3_	3.76, s	52.5, CH_3_	3.76, s
24			167.5, C		167.5, C	
25			131.4, C		131.4, C	
26			134.0, CH	6.82, td (1.5, 5.5)	133.9, CH	6.82, td (1.5, 5.5)
27			65.9, CH_2_	4.69, d (5.5)	65.9, CH_2_	4.68, d (5.5)
28			13.4, CH_3_	1.90, s	13.4, CH_3_	1.90, s

The molecular formula of compound **3** was determined as C_27_H_34_O_9_ by its HRESIMS ion peak (*m*/*z* 503.2279 [M + H]^+^), which corresponded to 11 units of unsaturations. A comparison of UV-vis and NMR data with those of **2** revealed that compound **3** also was an analogue of asteltoxin. The only difference was the absence of methylene between C-1 and C-3. This was proved by the HMBC spectrum showing correlation of H_3_-1 with C-3, C-4 and the splitting pattern of H_3_-1 (δ_H_ 1.17, d, *J* = 6.0 Hz) and H-3 (δ_H_ 4.57, q, *J* = 6.0 Hz). Based on the NOESY experiments, the relative configuration of **3** was also suggested to be analogous to that of an asteltoxin and named asteltoxin F [18].

**Figure 4:**
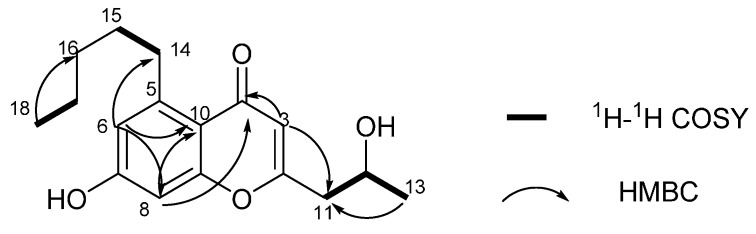
Selected ^1^H-^1^H COSY and HMBC correlations of **4**.

Compound **4** was obtained as needle crystal. The molecular formula of **4** was established as C_17_H_22_O_4_ by the HRESIMS ion peak at *m* /*z* 291.1588 [M + H]^+^ (calculated for C_17_H_23_O_4_, 291.1591) requiring seven degrees of unsaturation. The strong absorptions in the IR spectrum at 3354 and 1628 cm^−1^ showed the existence of a hydroxy group and a carbonyl group [19,20]. The ^1^H-NMR spectrum showed 20 proton signals, two sp^3^ aliphatic methy protons (δ_H_ 0.86 and 1.15), five sp^3^ methylene protons (δ_H_ 1.29, 1.29, 1.48, 2.57 and 3.08), one oxymethine proton (δ_H_ 4.03), and three sp^2^ methine protons (δ_H_ 5.95, 6.59 and 6.62). The ^13^C-NMR spectra showed 17 signals, comprising two methyls, five methylenes, four methines and six quaternary carbon atoms. Accounting for five of the seven degrees of unsaturation suggested the presence of two rings. The ^1^H- and ^13^C-NMR spectroscopic data of compound **4** indicate that it is a chromone analogue. The only difference from 5-carbomethoxy methyl-2 heptyl-7-hydroxy chromone was the substituent of C-5 [21]. The ^1^H-^1^H COSY correlation of H_2_-14/H_2_-15, H_2_-17/H_3_-18 and HMBC correlations of H_3_-18/C-16 and C-17, H_2_-15/C-16 and C-17 indicated the presence of a pentyl moiety. In addition, the HMBC correlation of H_3_-14/C-5, C-6 and C-10 suggested that the pentyl was located at C-5 (Figure 4 and Table 2).

**Table 2:** The ^1^H- and ^13^C-NMR Data for **4** (500/125 MHz, respectively in DMSO-*d*_6_, δ ppm, *J* in Hz).

Position	δ_H_ mult (*J* in Hz)	δ_C_	COSY	HMBC
1				
2		164.5, C		
3	5.95, s	111.6, CH	H-2	
4		177.8, C		
5		146.2, C		
6	6.59, d (2.0)	116.1, CH		C-7, 8, 10, 14
7		161.5, C		
8	6.62, d (2.0)	100.7, CH		C-4, 6, 7, 9, 10
9		159.5, C		
10		113.6, C		
11	2.57, m	42.8, CH_2_		C-2, 3, 12, 13
12	4.03, m	64.1, CH	H-11, H-13	C-2, 11
13	1.15, d (6.5)	23.5, CH_3_		C-11, 12
14	3.08, td (7.5, 4.5)	34.2, CH_2_	H-15	C-5, 6, 10, 15
15	1.48, m	30.8, CH_2_		C-5, 14, 16, 17
16	1.29, m	31.3, CH_2_		
17	1.29, m	22.0, CH_2_	H-18	C-16, 18
18	0.86, t (7.0)	14.0, CH_3_		C-16, 17

The absolute configuration of C-12 was established by comparison of the optical rotation of compound **4** ${\left[\alpha \right]}_{\text{D}}^{25}$ = +17.11 (*c* 0.31, MeOH) with that of 7-Hydroxy-2-(2-hydroxypropyl)-5-methyl chromone: ${\left[\alpha \right]}_{\text{D}}^{25}$ = +32 (*c* 0.02, MeOH) [21,22]. Thus, this establishes the absolute configuration of C-12 to be *S.* Thus, the structure of compound **4** was determined and named as 7-hydroxy-2-(2-hydroxypropyl)-5-pentylchromone.

### 2.2. Biological Activities

The antiviral (H1N1 and H3N2) activities of these compounds were individually evaluated through Cytopathic Effect (CPE) inhibition assay. Compounds **2** and **3** showed significant inhibitory activities against the H3N2 strain with the prominent IC_50_ values of 6.2 ± 0.08 and 8.9 ± 0.3 μM, respectively. In addition, compound **2** also exhibited significant activity against the H1N1 strain with an IC_50_ value of 3.5 ± 1.3 μM.

### 2.3. Characterization and Identification of Isolated Strain SCSIO XWS02F40

**Figure 5:**
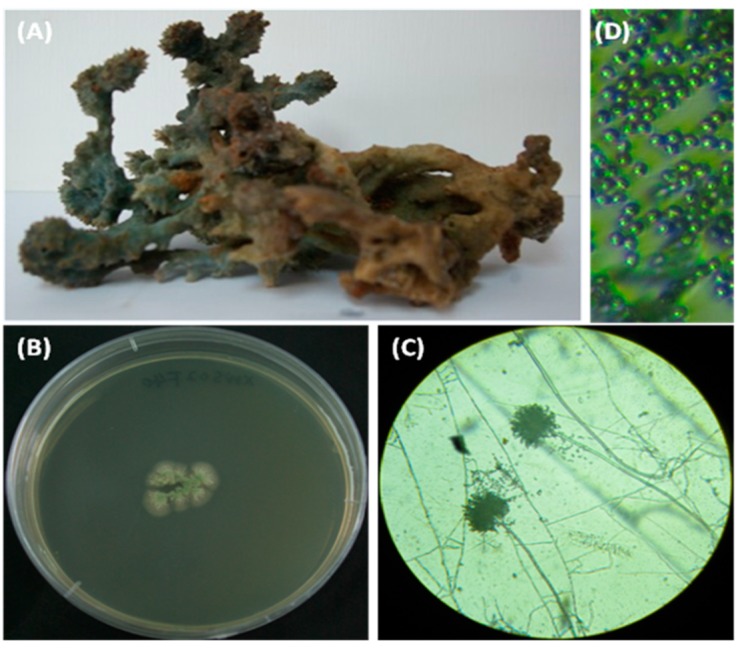
Colony appearance and micromorphology of *Aspergillus* sp. SCSIO XWS02F40 and the sample of the sponge *Callyspongia* sp. (**A**) sample of the sponge *Callyspongia* sp.; (**B**) colony appearance after seven days at 25 °C (MB medium); (**C**) conidiophores after seven days at 25 °C under a light microscope; (**D**) conidia as seen using a light microscope.

The fungal strain SCSIO XWS02F40, which was isolated from a sponge *Callyspongia* sp. collected from the sea area near Xuwen County, Guangdong Province, China, exhibited potential antiviral activity in our previous screening tests. After seven days of growth on MB (Malt Extract Agar Base) medium at 25 °C, colonies were 15 mm to 20 mm in diameter, showed good sporulation, and were light green, whereas the color of the reverse was the same as that of the surface (Figure 5B). Light microscopy revealed that the stipes were erecting hyphae. Spherical expansion (vesicles) were formed at the end of the stipes, on which there were green conidial chains. A teleomorphic state was not observed (Figure 5C,D).

**Figure 6:**
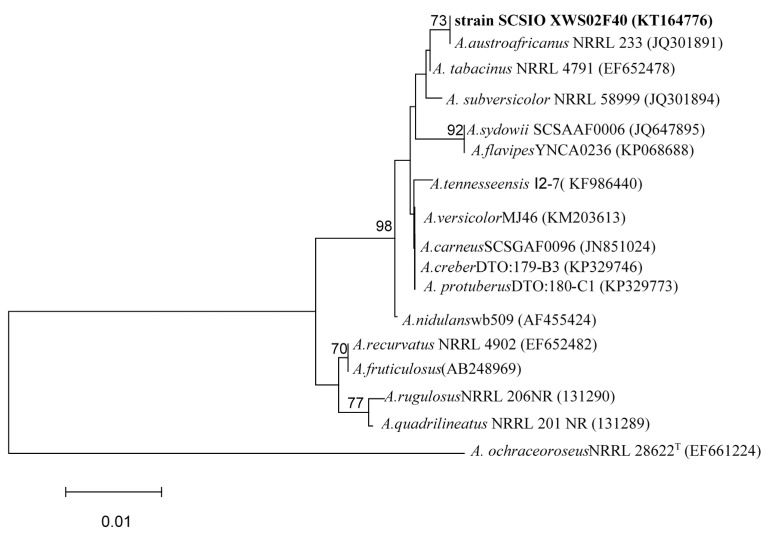
The neighbor-joining tree based on ITS1-5.8S-ITS2 sequences, showing a phylogenetic relationship between strain SCSIO XWS02F40 and related *Aspergillus* species. Only boot strap values >70% are shown. GenBank accession numbers are given in *parentheses*. Bar: 1% sequence divergence.

The ITS1-5.8S-ITS2 sequence region (524 base pairs (bp), accession number KT164776) of strain SCSIO XWS02F40 was amplified by PCR and sequenced. A phylogenetic tree was procured by the neighbor-joining method as per a similarity based off a 510-bp consensus length of the ITS1-5.8S-ITS2 sequence (Figure 6). Strain SCSIO XWS02F40 was found to belong to a clade related to *A.* *austroafricanus* NRRL 233 (accession number JQ301891), with a sequence identity of 99.4%. On the basis of its properties of culture and morphology, and ITS phylogenetic analysis, strain SCSIO XWS02F40 was identified as a member of the *Aspergillus* genus, and was named as *Aspergillus* sp. SCSIO XWS02F40.

## 3. Experimental Section

### 3.1. General Experimental Procedures

The NMR spectra were measured on a Bruker AC 500 MHz NMR (Bruker, Fällanden, Switzerland) spectrometer with TMS as an internal standard. High resolution mass spectra (HRESIMS) were recorded on a Bruker micro TOF-QII mass spectrometer (Bruker, Fällanden, Switzerland). CD spectra were measured with a Chirascan circular dichroism spectrometer (Applied Photophysics, Surrey, UK). Size exclusion chromatography was done on Sephadex LH-20 gel (GE Healthcare, Uppsala, Sweden). Column chromatography (CC) was carried out on silica gel (200–300 mesh); (Qingdao Marine Chemical Factory, Qingdao, China). Spots were detected on TLC under UV light or by heating after spraying with 12% H_2_SO_4_ and enough vanillin in H_2_O.

### 3.2. Fungal Materials

The fungal strain SCSIO XWS02F40 was isolated from the sponge *Callyspongia* sp., which was collected from the sea area near Xuwen County, Guangdong Province, China, during August 2013. The isolate was stored on MB agar (malt extract 15 g, sea salt 10 g, agar 15 g) slants at 4 °C and then deposited at CAS Key Laboratory of Tropical Marine Bio-resources and Ecology.

### 3.3. Cultural and Morphological Properties of Strain SCSIO XWS02F40

The cultural properties and the morphological features of the spores and mycelia of strain SCSIO XWS02F40 were examined on MB agar medium after culturing at 25 °C for 7 day. The samples were observed with a B204 series biological microscope (Chongqing Optec Instrument Co., Ltd., Chongqing, China) light microscope using a previously described cover technique.

### 3.4. ITS Region Sequence and Phylogenetic Analysis

The mycelia of strain SCSIO XWS02F40 cultured in Sabouraud’s Dextrose Broth (consisting of 40 g dextrose, 10 g peptone, 2.5 g NaCl, and 1000 mL distilled water, pH 5.6) were sampled and powdered in a mixer mill after liquid nitrogen was added. DNA was isolated through the Hpure Fungal DNA Kit (Guangzhou Genebase Bioscience Co., Guangzhou, China) according to the manufacturer’s protocol. The ITS region of strain SCSIO XWS02F40 was amplified by polymerase chain reaction with the primer pair ITS1–ITS4. The amplified product was purified with a TIANgel mini purification kit (TianGen Biotech, Beijing, China). Pure PCR product was submitted for sequencing together with the primer ITS1 to a commercial service (Shanghai Majorbio Bio-pharm Technology Co., Ltd., ShangHai, China). The derived ITS region sequence was compared against the GenBank database (NCBI) through BLAST-Algorithmus. Similarity analysis was performed using Clustal W program. The phylogenetic tree of strain SCSIO XWS02F40 was constructed using neighbor-joining method. *A.* *ochraceoroseus* NRRL 28622^T^was used as an out group.

### 3.5. Nucleotide Sequence Accession Number

The nucleotide sequence of the ITS region reported in this article was assigned the GenBank accession number KT164776.

### 3.6. Fermentation and Extraction

*Aspergillus* sp. was cultured on MB-agar plates at 25 °C for seven days. The seed medium consisted of malt extract: 15 g, sea salt: 10 g, distilled water: 1000 mL, pH 7.4–7.8, and was inoculated with strain SCSIO XWS02F40 and incubated at 25 °C for 72 h on a rotating shaker (170 rpm). Mass scale fermentation of fungal isolate SCSIO XWS02F40 was carried out using solid rice medium in 1000 mL flasks (rice: 200 g, sea salt: 2.5 g, distilled water: 200 mL), and inoculated with 10 mL of seed solution. Flasks were incubated at 25 °C under normal day-night cycle. After 30 days, cultures from 30 flasks were harvested and subjected for organic extraction using Ethyl acetate (EtOAc). The EtOAc extracts of rice solid media of *Aspergillus* sp. SCSIO XWS02F40 were partitioned between petroleum ether, and 90% aqueous MeOH, The resulting MeOH phase was fractionated using silica column, Sephadex LH-20, and then semi-preparative reversed-phase HPLC to yield compounds **1**–**7** (Figure 1).

The culture of solid rice medium was soaked in acetone and cut into small pieces and kept for 1 day. The content was filtered and evaporated under vacuum using a Buchner funnel and extracted with EtOAc until exhaustion and this process was repeated thrice. The organic phase was collected and evaporated to obtain a dark brown oil crude extract (60.5 g). The crude EtOAc extract was subjected to silica gel column chromatography (CC) eluted with petroleum ether/ EtOAc in a gradient eluent (*v*/*v*, 50:1, 30:1, 20:1, 10:1, 5:1, 1:1, 0:1) to obtain 10 fractions (fractions 1–10) on the basis of TLC. Fr. 3 (5.7 g) was purified by Sephadex LH-20 (CHCl_3_/MeOH, 1:1) to afford compound **5** (4.7 g). Fr. 4 (118.6 mg) was purified by semi-preparative reversed-phase (SP-RP) HPLC using a C18 column (YMC-Pack, ODS-A S-5 μm × 12 nm 250 × 20 mm i.d., 4 mL/min) eluting with MeOH/H_2_O (70:30) to afford compound **6** (8.0 mg). Fr.5 (1.6 g) was purified by Sephadex LH-20 (CHCl_3_/MeOH, 1:1) yielding three sub-fractions (fr. 5.1–5.3). Fr. 5.2 (400 mg) was further purified by (SP-RP) HPLC eluting with CH_3_OH-H_2_O (65:35) to afford compounds **1** (5.5 mg), **2** (25.3 mg), and **3** (12.0 mg). Fr.7 (410 mg) was subjected to ODS chromatography eluted with MeOH/H_2_O in a gradient eluent (1:9, 2:3, 3:2, 4;1, 9:1), to give 4 sub fractions (fr. 7.1–7.4). Fr. 7.1 (90 mg) was further purified by (SP-RP) HPLC eluting with CH_3_CN-H_2_O (45:55) to afford compounds **4** (7.8 mg) and **7** (8.9 mg).

*Asteltoxin* *E* (**2**): Yellow amorphous solid; ${\left[\alpha \right]}_{\text{D}}^{25}$ +21.4 (*c* 0.72, MeOH); UV (MeOH) *λ* max (log ε) 356 (2.87), 300 (2.74), 267 (3.05), 211 (3.27) nm; FT-IR: 3390, 1697, 1002 cm^−^^1^; ^1^H-and ^13^C-NMR data, see Table 1; HRESIMS *m*/*z* 517.2440 [M + H]^+^ (calcd for C_28_H_37_O_9_, 517.2432).

*Asteltoxin* *F*(**3**): Yellow amorphous solid; ${\left[\alpha \right]}_{\text{D}}^{25}$ +20.4 (*c* 0.48, MeOH); UV (MeOH) *λ* max (log ε) 356 (2.90), 300 (2.53), 267 (3.11), 210 (3.26) nm; FT-IR: 3333, 1695, 1013 cm^−^^1^; ^1^H- and ^13^C-NMR data, see Table 1; HRESIMS *m*/*z* 503.2279 [M + H]^+^ (calcd for C_27_H_35_O_9_, 503.2276).

*7-Hydroxy-2-(2-hydroxypropyl)-5-pentylchromone* (**4**): Dark red needle crystals; ${\left[\alpha \right]}_{\text{D}}^{25}$ +17.11 (*c* 0.31, MeOH): UV (MeOH) *λ* max (log ε) 292 (3.72), 251 (3.86), 239 (3.84), 210 (4.04) nm; FT-IR: 3354, 1628, 1014 cm^−^^1^; ^1^H- and ^13^C-NMR data, see Table 2; HRESIMS *m*/*z* 291.1588 [M + H]^+^ (calcd for C_17_H_23_O_4_, 291.1591).

### 3.7. Bioassay Protocols

The antiviral activities against H1N1 and H3N2 were evaluated by the CPE inhibition assay in duplicate assay [23]. Confluent MDCK cell monolayers were firstly incubated with influenza virus at 37 °C for 1 h. After removing the virus dilution, cells were maintained in infecting media (RPMI 1640, 4 μg/mL of trypsin) containing different concentrations of test compounds. After 48 h incubation at 37 °C, the cells were fixed with 100 μL of 4% formaldehyde for 20 min at room temperature. After removal of the formaldehyde, the cells were stained with 0.1% crystal violet for 30 min. The plates were washed and dried, and the intensity of crystal violet staining for each well was measured in a microplate reader (Bio-Rad, Hercules, CA, USA) at 570 nm. The IC_50_ was calculated as the compounds concentration required to inhibit influenza virus yield at 48 h post-infection by 50%. Oseltamivir was used as the positive control with IC_50_ values of 18.5 and 16.9 nM, respectively.

## 4. Conclusions

In conclusion, three new compounds, Asteltoxin E (**2**), Asteltoxin F (**3**) and 7-hydroxy-2-(2-hydroxypropyl)-5-pentyl chromone (**4**), together with four known compounds were isolated from a marine sponge–derived fungus, *Aspergillus* sp. Compounds **2** and **3** are analogues of asteltoxin. Compound **4** is a new chromone derivative and its pentylbenzene moiety is a rare incidence in nature. Compound **5** is a liver carcinogen and forms DNA adducts after metabolic activation to an epoxide at the furofuran ring [24]. It is noteworthy that compound **5** was obtained in a scale of 4.7 g and a new dimensional pharmacokinetic research of this compound is a part of our ongoing mission. In the screening to search for seeds of antiviral agents, compounds **2** and **3** showed significant activity against H3N2 with the prominent IC_50_ value of 6.2 ± 0.08 and 8.9 ± 0.3 μM; respectively. In addition; compound **2** also exhibited inhibitory activity against H1N1 with IC_50_ value of 3.5 ± 1.3 μM. Compounds **2** and **3** were published online on 30 November 2015 as new compounds after the date we submitted on 2 November 2015 [25].

## Supplementary Materials

Supplementary materials can be accessed at: http://www.mdpi.com/1420-3049/21/1/34/s1.
