# Supplementary material for: Asteltoxins with Antiviral Activities from the Marine Sponge-Derived Fungus Aspergillus sp. SCSIO XWS02F40
Source: Molecules. 2015 Dec 26;21(1):34. doi: 10.3390/molecules21010034 (PMC6272915; doi:10.3390/molecules21010034)
Supplement: Supplementary file 1 [file molecules-21-00034-s001.pdf]

Supplementary Materials: Asteltoxins with Antiviral Activities from the Marine Sponge-Derived Fungus *Aspergillus* sp. SCSIO XWS02F40

Yong-Qi Tian [1,2], Xiu-Ping Lin [1], Zhen Wang [3], Xue-Feng Zhou [1], Xiaochu Qin [3], Kumaravel Kaliyaperumal [1], Tianyu Zhang [3], Zheng-Chao Tu [3] and Yonghong Liu [1,*]

Contents

(1) **Figure S1.** ^{1}H-NMR spectrum of compound **2**(500 MHz, $CDCl_3$).
(2) **Figure S2.** ^{13}C-NMR spectrum of compound **2** (125 MHz, $CDCl_3$).
(3) **Figure S3.** DEPT-135 spectrum of compound **2** (125 MHz, $CDCl_3$).
(4) **Figure S4.** HSQC spectrum of compound **2** (500 MHz, $CDCl_3$).
(5) **Figure S5.** COSY spectrum of compound **2** (500 MHz, $CDCl_3$).
(6) **Figure S6.** HMBC spectrum of compound **2** (500 MHz, $CDCl_3$).
(7) **Figure S7.** NOE spectrum of compound **2** (600 MHz, $CDCl_3$).
(8) **Figure S8.** HRESIMS spectrum of compound **2**.
(9) **Figure S9.** ^{1}H-NMR spectrum of compound **3** (500 MHz, $CDCl_3$).
(10) **Figure S10.** ^{13}C-NMR spectrum of compound **3** (125 MHz, $CDCl_3$).
(11) **Figure S11.** DEPT-135 spectrum of compound **3** (125 MHz, $CDCl_3$).
(12) **Figure S12.** HSQC spectrum of compound **3** (500 MHz, $CDCl_3$).
(13) **Figure S13.** COSY spectrum of compound **3**(500 MHz, $CDCl_3$).
(14) **Figure S14.** HMBC spectrum of compound **3** (500 MHz, $CDCl_3$).
(15) **Figure S15.** NOE spectrum of compound **3** (600 MHz, $CDCl_3$).
(16) **Figure S16.** HRESIMS spectrum of compound **3**.
(17) **Figure S17.** ^{1}H-NMR spectrum of compound **4** (500 MHz, DMSO-d_6).
(18) **Figure S18.** ^{13}C-NMR spectrum of compound **4** (125 MHz, DMSO-d_6).
(19) **Figure S19.** DEPT-135 spectrum of compound **4** (125 MHz, DMSO-d_6).
(20) **Figure S20.** HSQC spectrum of compound **4** (500 MHz, DMSO-d_6).
(21) **Figure S21.** COSY spectrum of compound **4** (500 MHz, DMSO-d_6).
(22) **Figure S22.** HMBC spectrum of compound **4** (500 MHz, DMSO-d_6).
(23) **Figure S23.** HRESIMS spectrum of compound **4**.

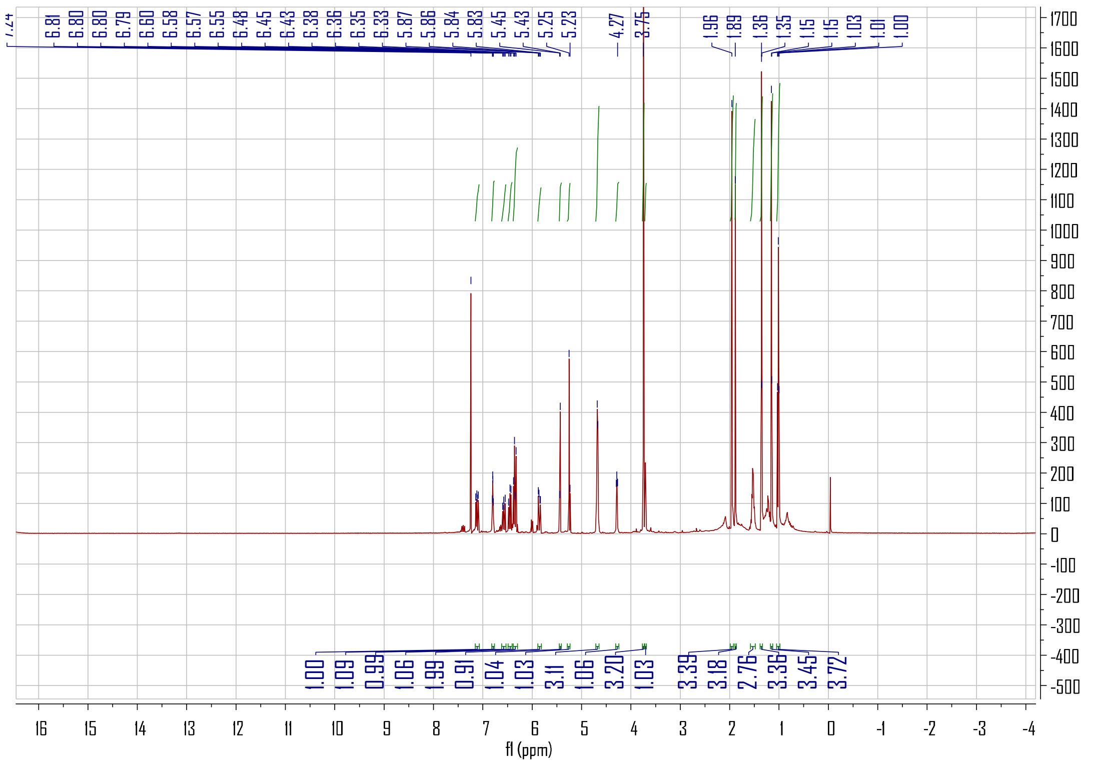

Figure S1. ^{1}H-NMR spectrum of compound **2** (500 MHz, $CDCl_3$).

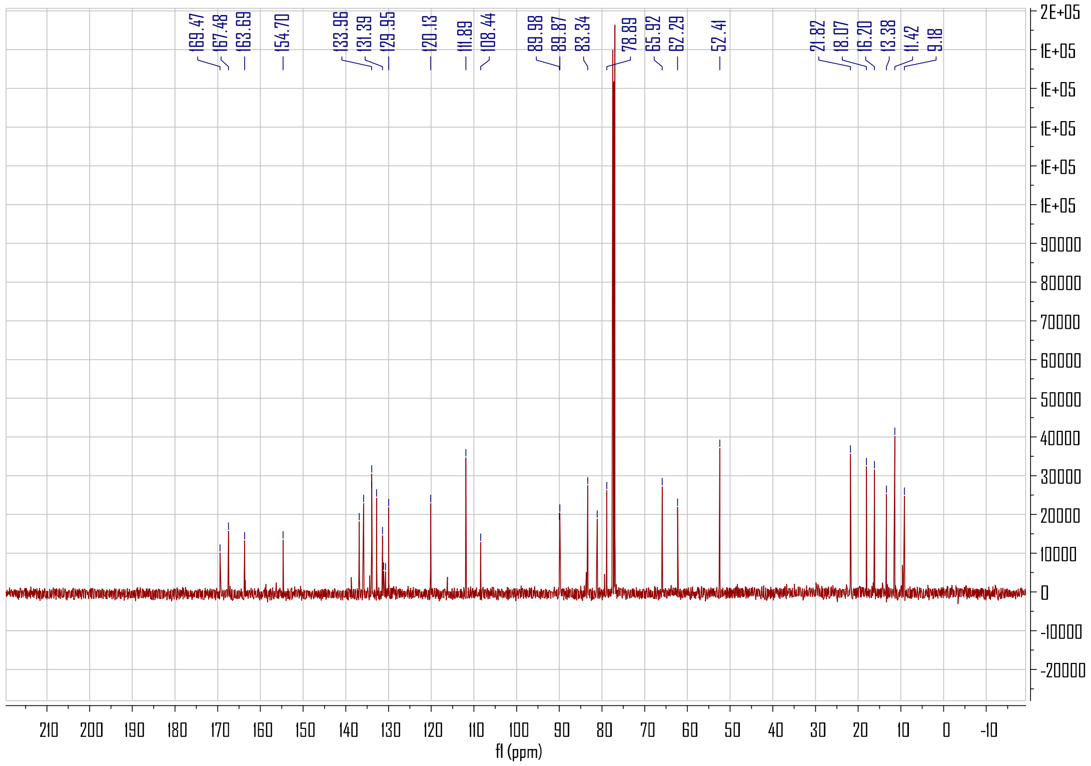

Figure S2. ^{13}C-NMR spectrum of compound **2** (125 MHz, $CDCl_3$).

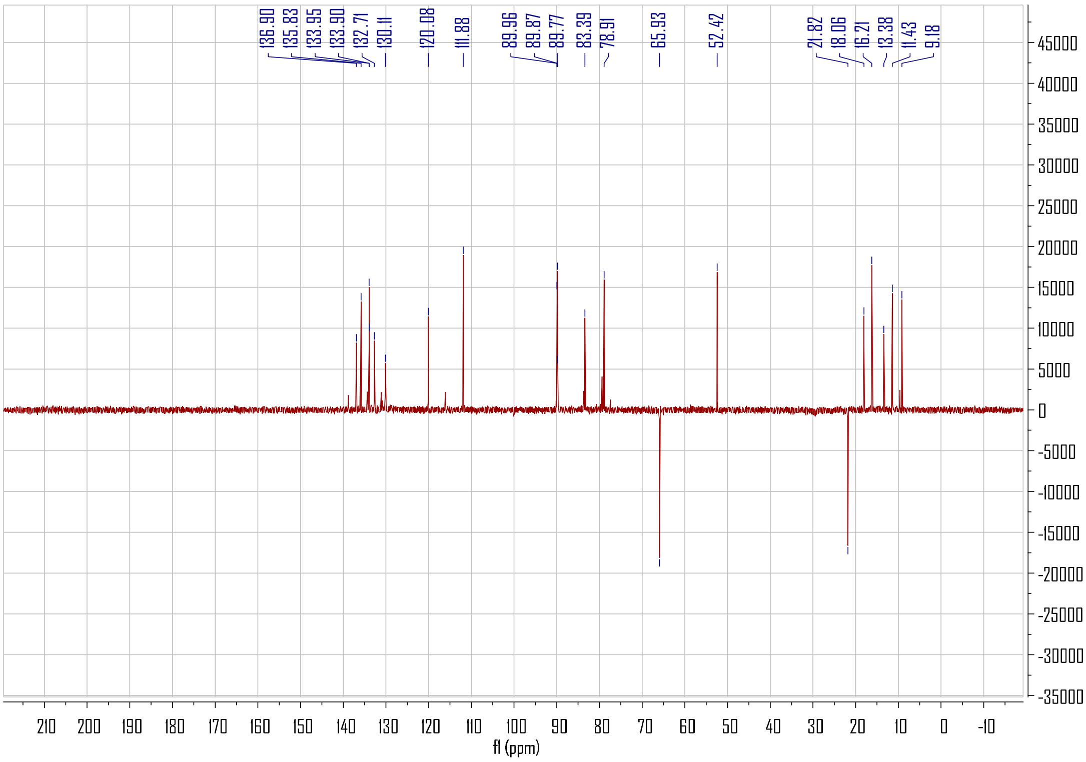

Figure S3. DEPT-135 spectrum of compound **2** (125 MHz, $CDCl_3$).

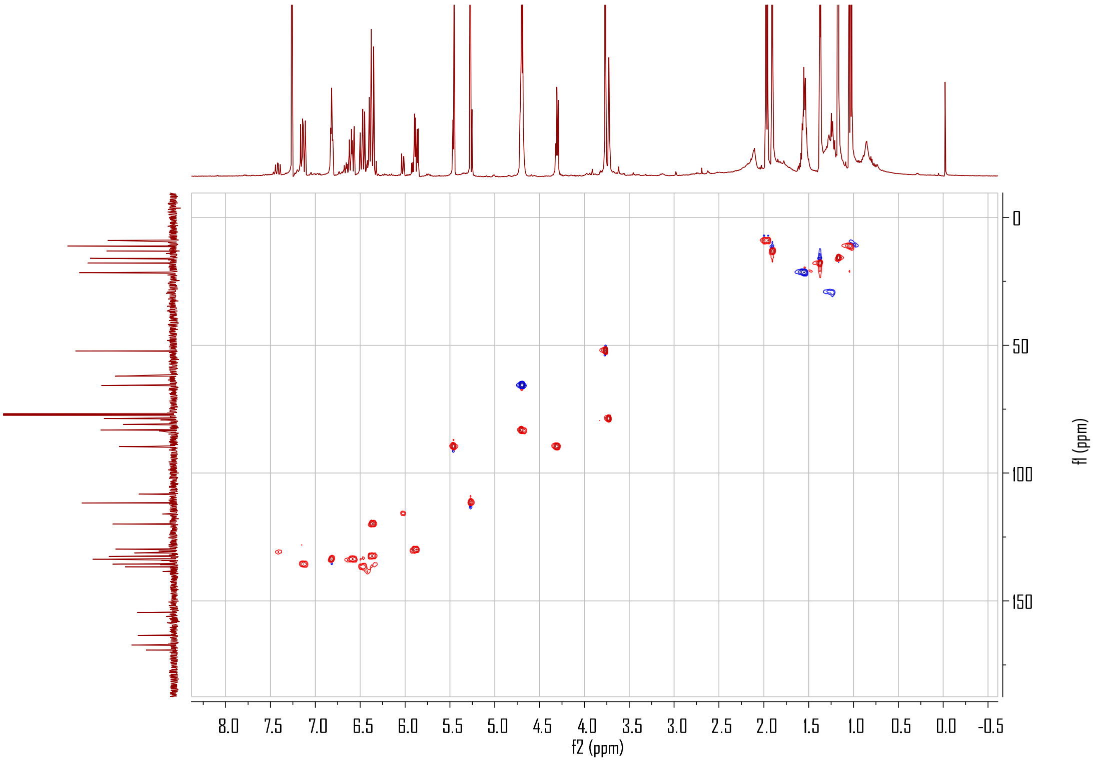

Figure S4. HSQC spectrum of compound **2** (500 MHz, $CDCl_3$).

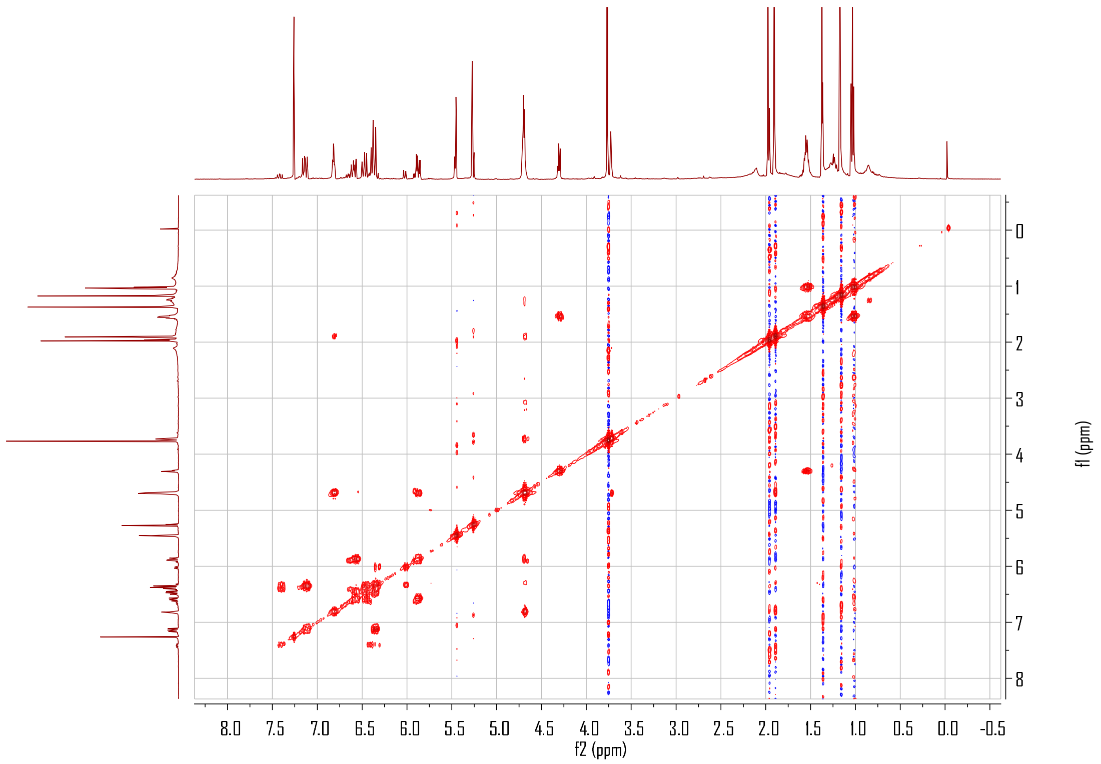

Figure S5. COSY spectrum of compound **2** (500 MHz, CDCl3).

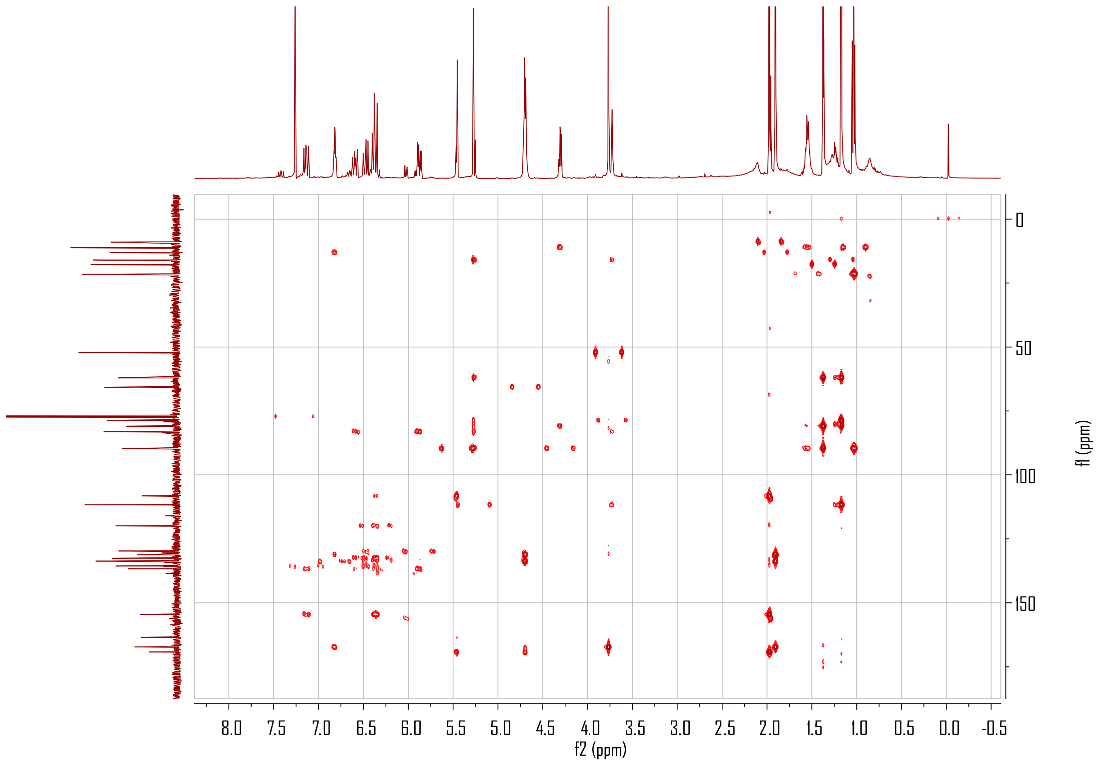

Figure S6. HMBC spectrum of compound **2** (500 MHz, CDCl3).

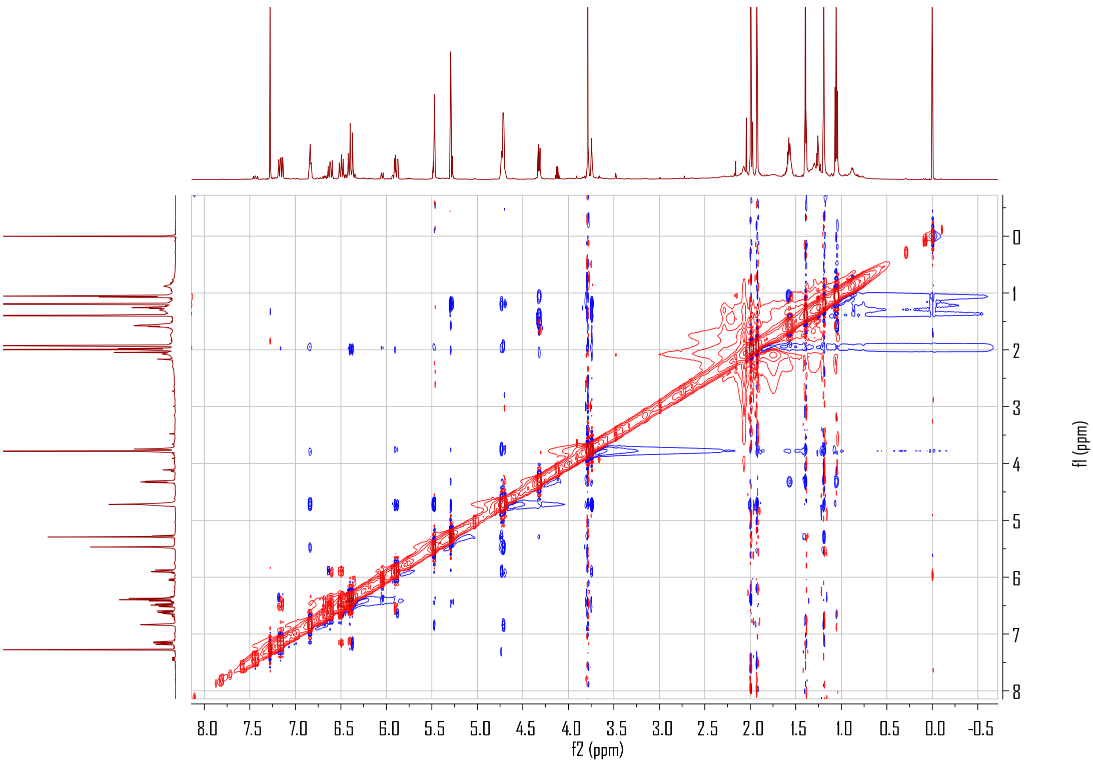

Figure S7. NOE spectrum of compound **2** (600 MHz, $CDCl_3$).

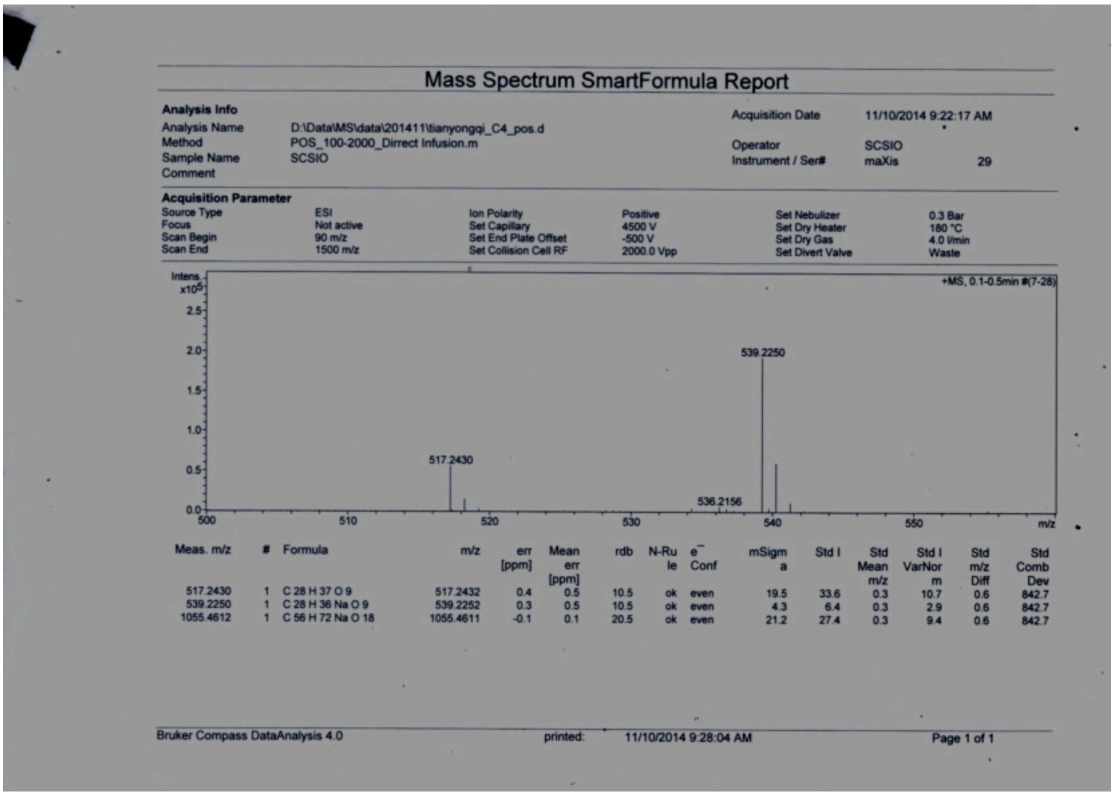

Mass Spectrum SmartFormula Report

Analysis Info

Analysis Name D:\Data\MS\data\201411\tianyongqi_C4_pos.d
Method POS_100-2000_Dirrect Infusion.m
Sample Name SCSIO
Comment

Acquisition Date 11/10/2014 9:22:17 AM
Operator SCSIO
Instrument / Ser# maXis 29

Acquisition Parameter

Source Type	ESI	Ion Polarity	Positive	Set Nebulizer	0.3 Bar
Focus	Not active	Set Capillary	4500 V	Set Dry Heater	180 °C
Scan Begin	90 m/z	Set End Plate Offset	-500 V	Set Dry Gas	4.0 l/min
Scan End	1500 m/z	Set Collision Cell RF	2000.0 Vpp	Set Divert Valve	Waste

Meas. m/z	#	Formula	m/z	err [ppm]	Mean err [ppm]	rdb	N-Rule	e⁻ Conf	mSigma	Std I	Std Mean m/z	Std I VarNorm	Std m/z Diff	Std Comb Dev
517.2430	1	C 28 H 37 O 9	517.2432	0.4	0.5	10.5	ok	even	19.5	33.6	0.3	10.7	0.6	842.7
539.2250	1	C 28 H 36 Na O 9	539.2252	0.3	0.5	10.5	ok	even	4.3	6.4	0.3	2.9	0.6	842.7
1055.4612	1	C 56 H 72 Na O 18	1055.4611	-0.1	0.1	20.5	ok	even	21.2	27.4	0.3	9.4	0.6	842.7

Bruker Compass DataAnalysis 4.0 printed: 11/10/2014 9:28:04 AM Page 1 of 1

Figure S8. HRESIMS spectrum of compound **2**.

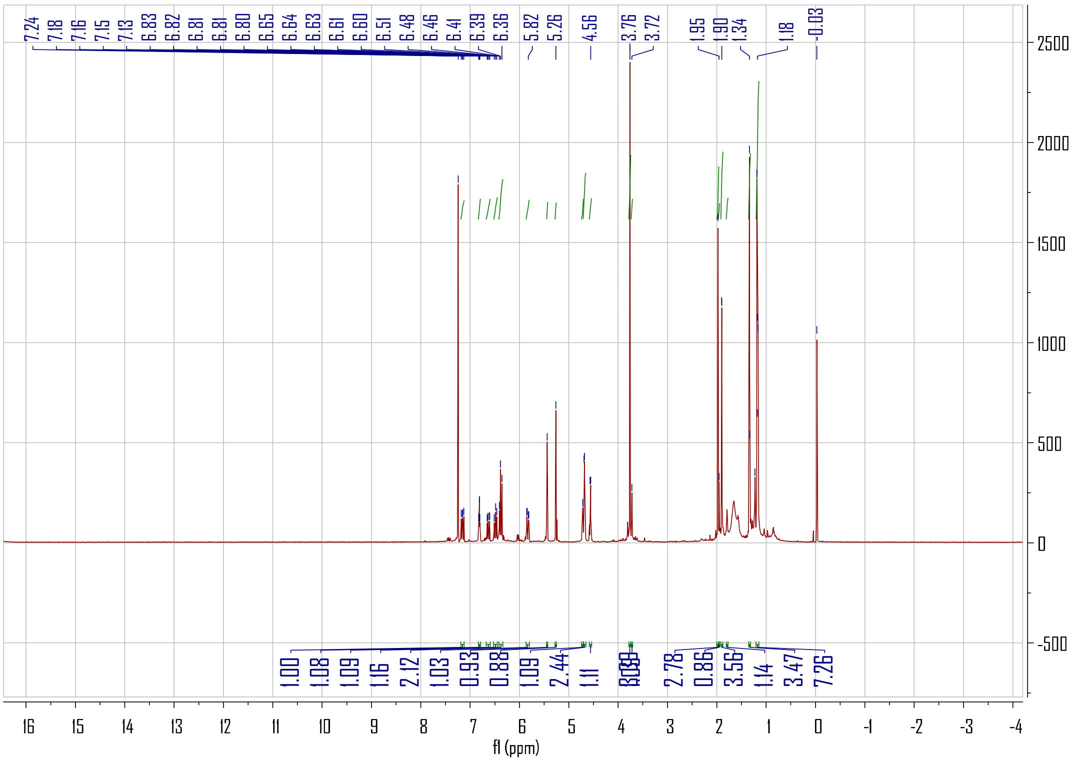

Figure S9. ^{1}H-NMR spectrum of compound **3** (500 MHz, $CDCl_3$).

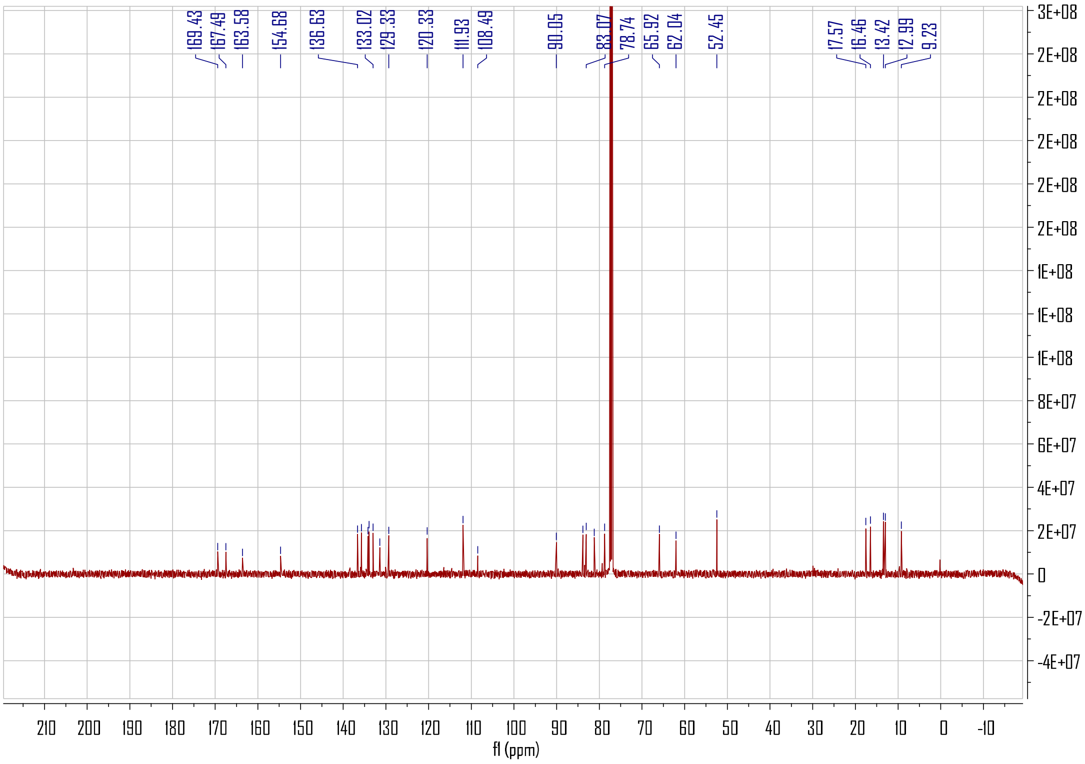

Figure S10. ^{13}C-NMR spectrum of compound **3** (125 MHz, $CDCl_3$).

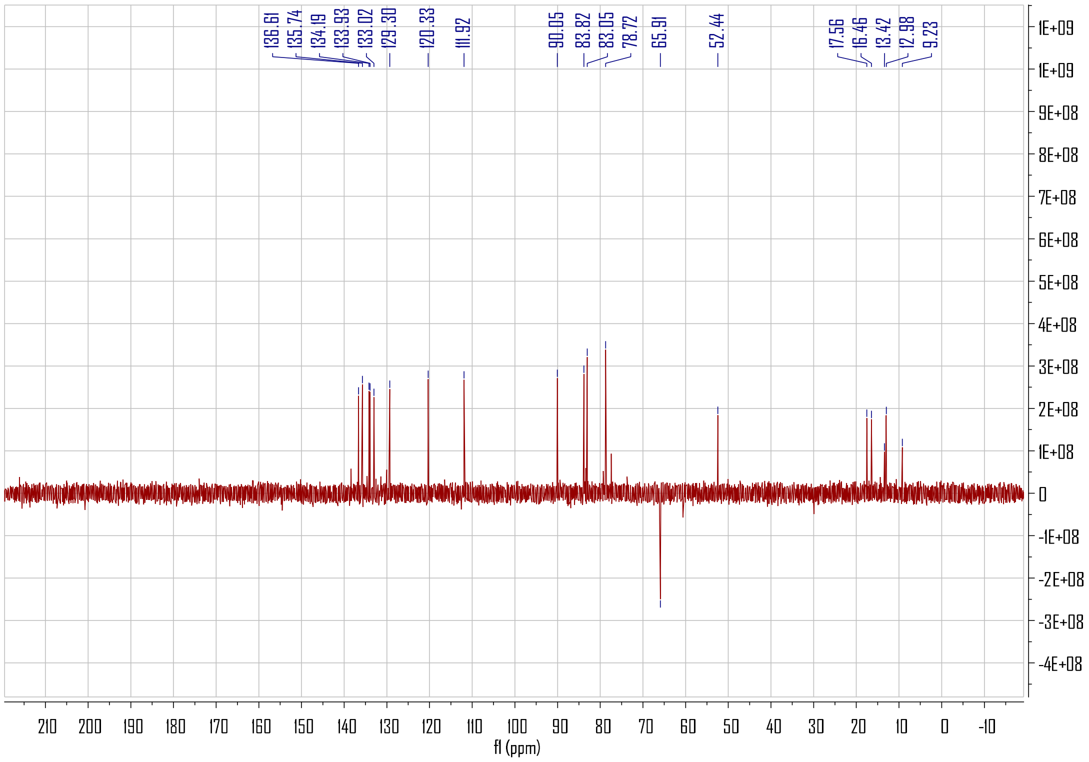

Figure S11. DEPT-135 spectrum of compound **3** (125 MHz, $CDCl_3$).

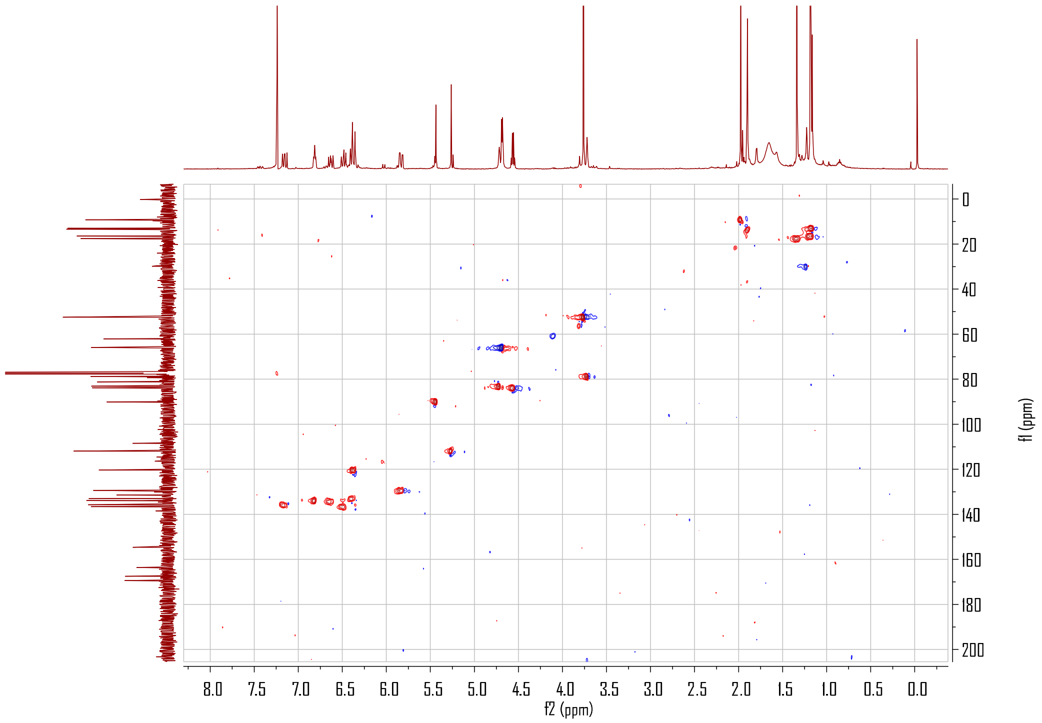

Figure S12. HSQC spectrum of compound **3** (500 MHz, $CDCl_3$).

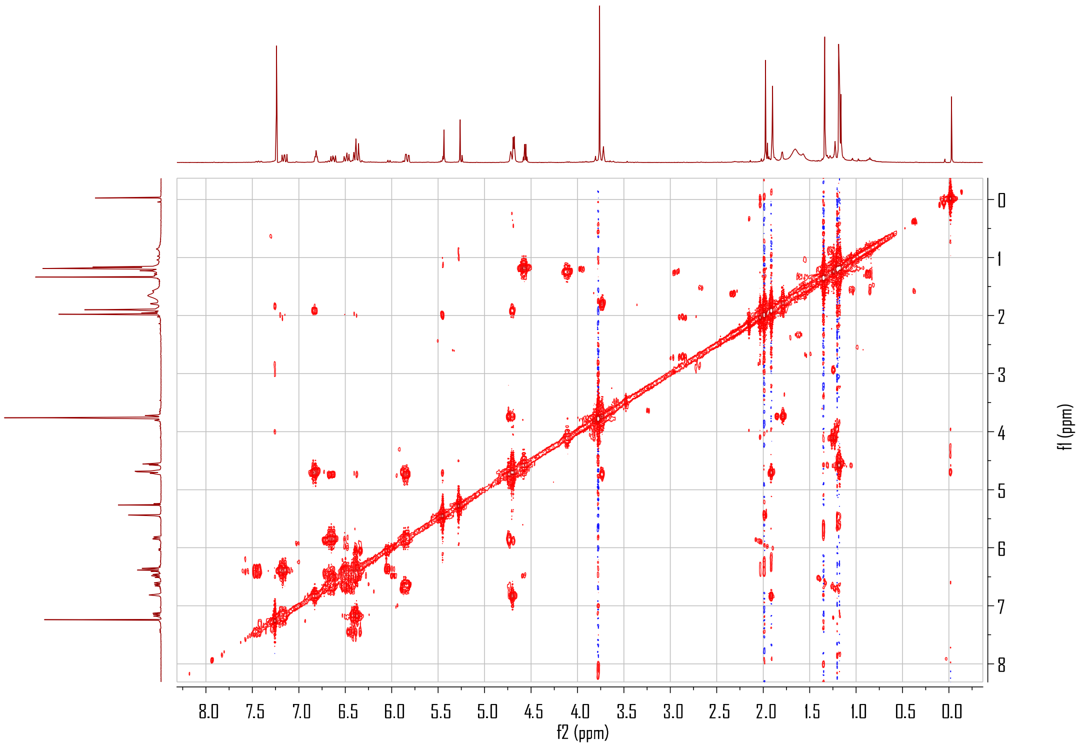

Figure S13. COSY spectrum of compound **3** (500 MHz, $CDCl_3$).

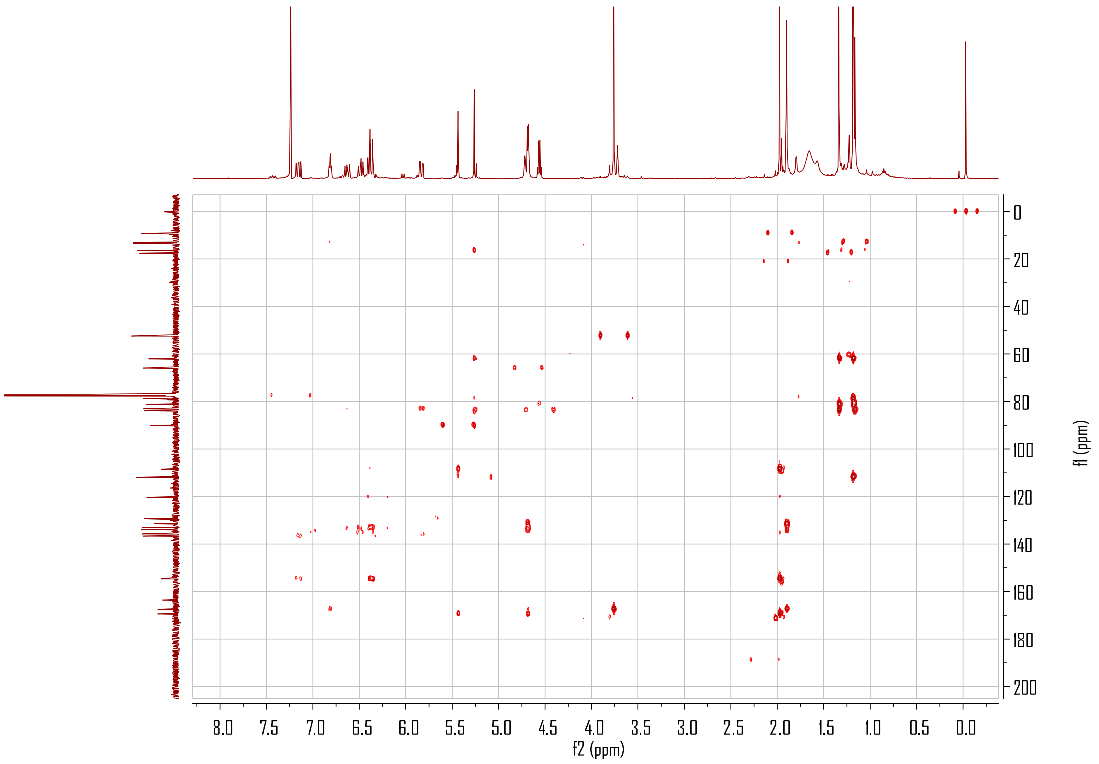

Figure S14. HMBC spectrum of compound **3** (500 MHz, $CDCl_3$).

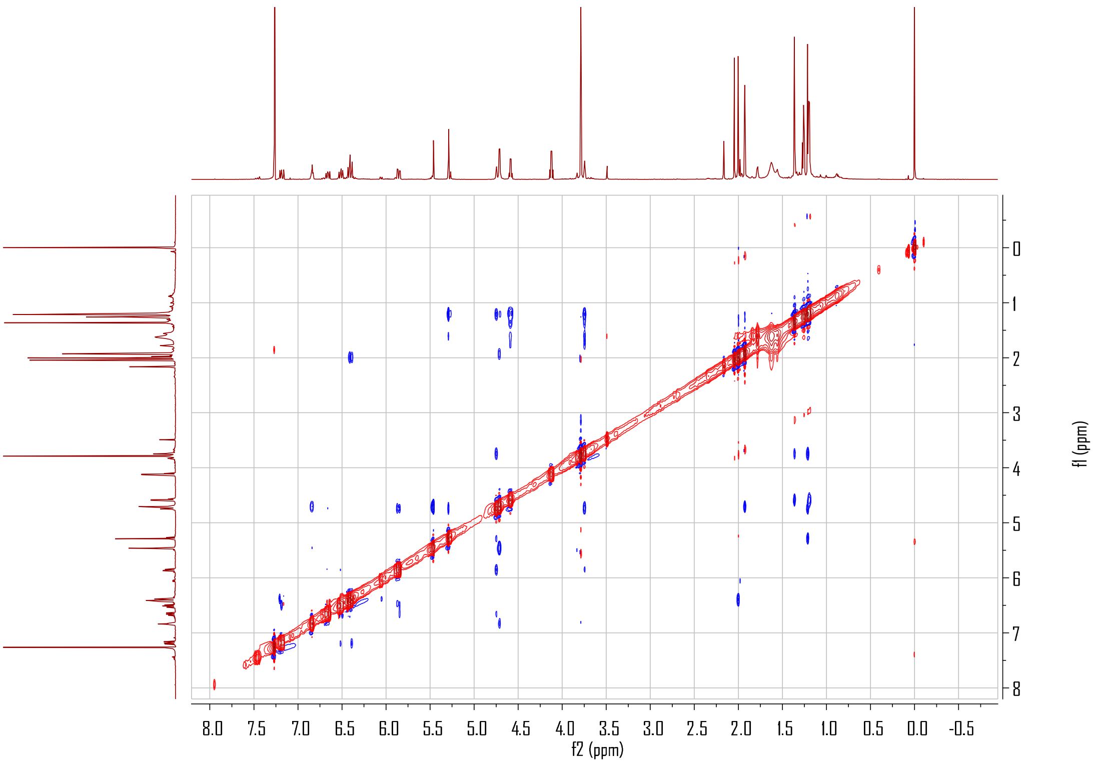

Figure S15. NOE spectrum of compound **3** (600 MHz, $CDCl_3$).

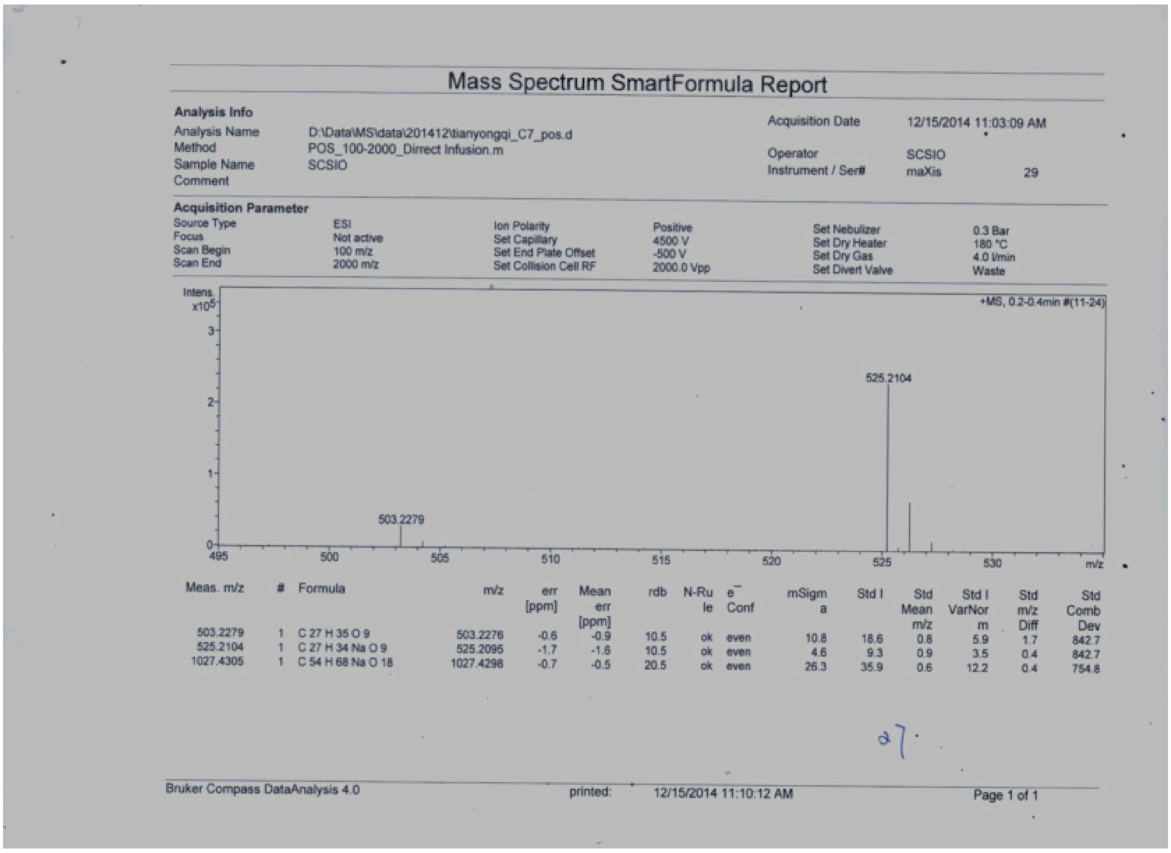

Mass Spectrum SmartFormula Report

Analysis Info

Analysis Name D:\Data\MS\data\201412\tianyongqi_C7_pos.d
Method POS_100-2000_Dirrect Infusion.m
Sample Name SCSIO
Comment

Acquisition Date 12/15/2014 11:03:09 AM
Operator SCSIO
Instrument / Ser# maXis 29

Acquisition Parameter

Source Type	ESI	Ion Polarity	Positive	Set Nebulizer	0.3 Bar
Focus	Not active	Set Capillary	4500 V	Set Dry Heater	180 °C
Scan Begin	100 m/z	Set End Plate Offset	-500 V	Set Dry Gas	4.0 l/min
Scan End	2000 m/z	Set Collision Cell RF	2000.0 Vpp	Set Divert Valve	Waste

Meas. m/z	#	Formula	m/z	err [ppm]	Mean err [ppm]	rdb	N-Rule	e⁻ Conf	mSigma	Std I	Std Mean m/z	Std I VarNorm	Std m/z Diff	Std Comb Dev
503.2279	1	C 27 H 35 O 9	503.2276	-0.6	-0.9	10.5	ok	even	10.8	18.6	0.8	5.9	1.7	842.7
525.2104	1	C 27 H 34 Na O 9	525.2095	-1.7	-1.6	10.5	ok	even	4.6	9.3	0.9	3.5	0.4	842.7
1027.4305	1	C 54 H 68 Na O 18	1027.4298	-0.7	-0.5	20.5	ok	even	26.3	35.9	0.6	12.2	0.4	754.8

a7.

Bruker Compass DataAnalysis 4.0 printed: 12/15/2014 11:10:12 AM Page 1 of 1

Figure S16. HRESIMS spectrum of compound **3**.

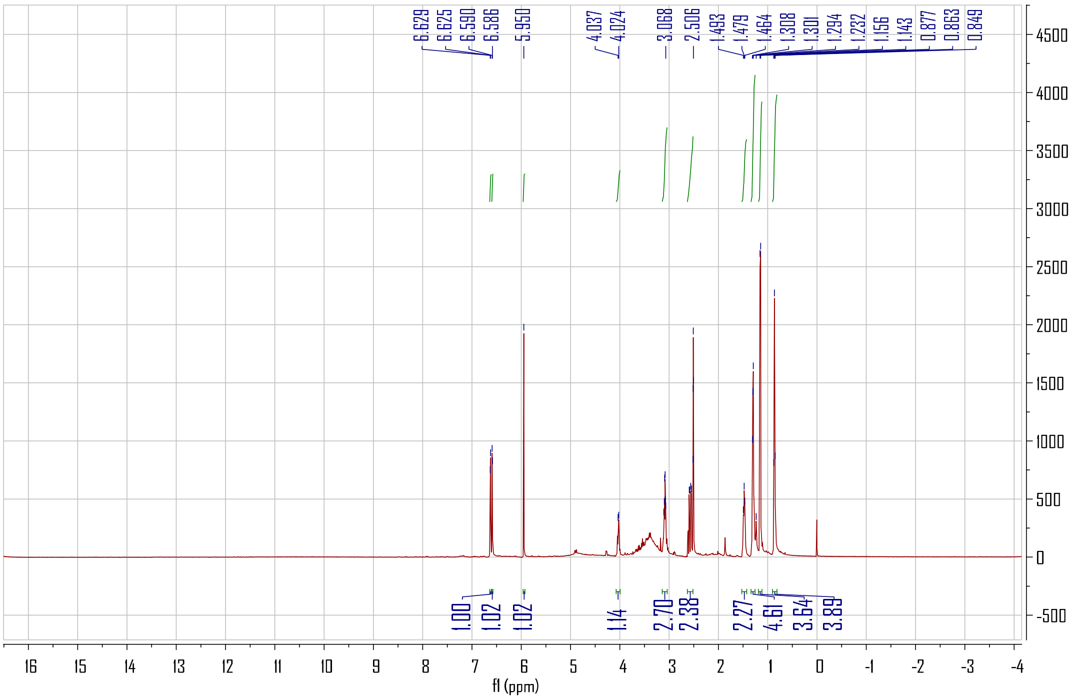

Figure S17. ^{1}H-NMR spectrum of compound **4** (500 MHz, DMSO-d_6).

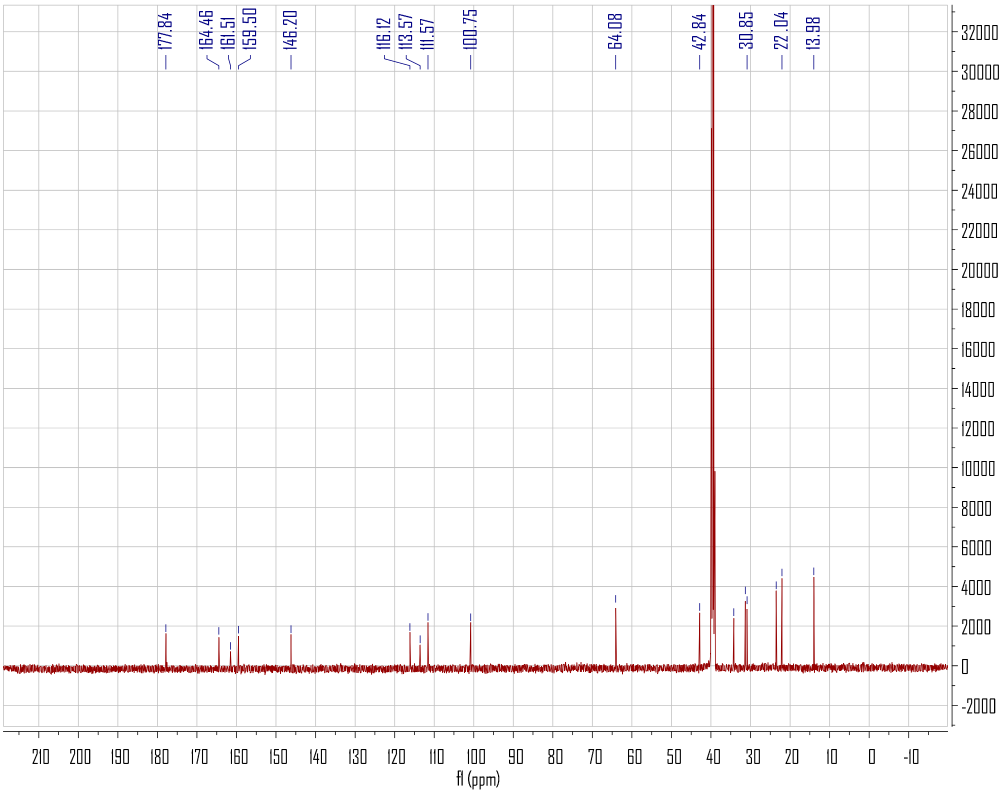

Figure S18. ^{13}C-NMR spectrum of compound **4** (125 MHz, DMSO-d_6).

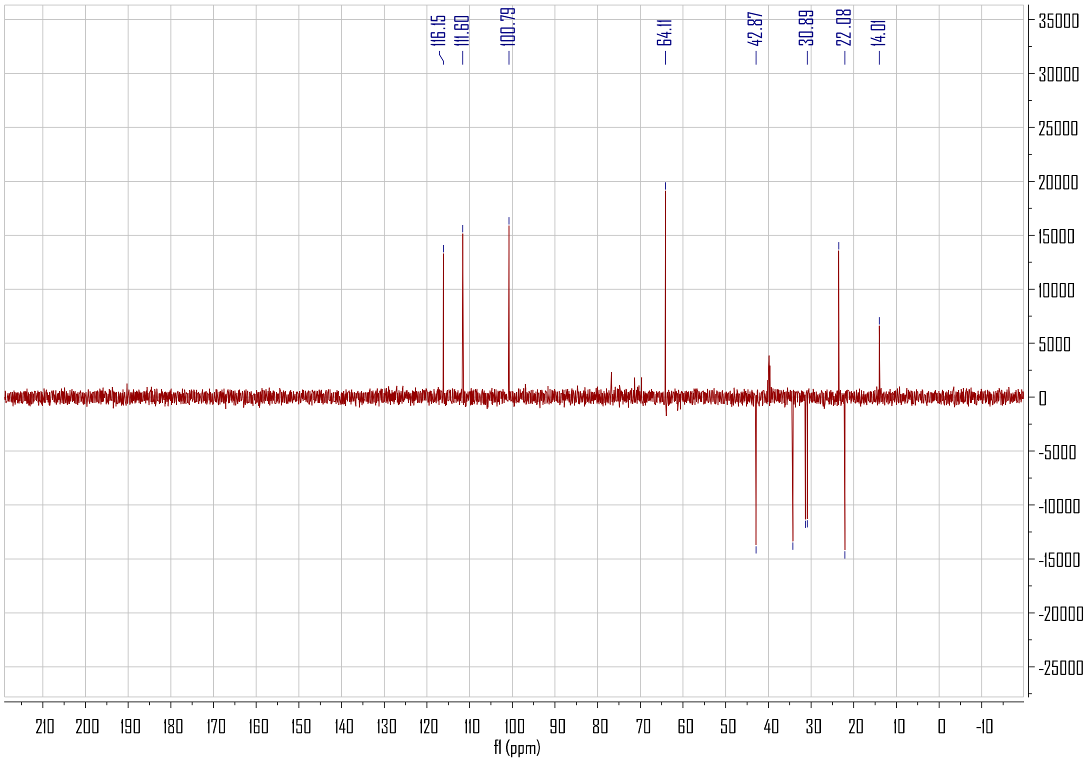

Figure S19. DEPT-135 spectrum of compound **4** (125 MHz, DMSO-*d*6).

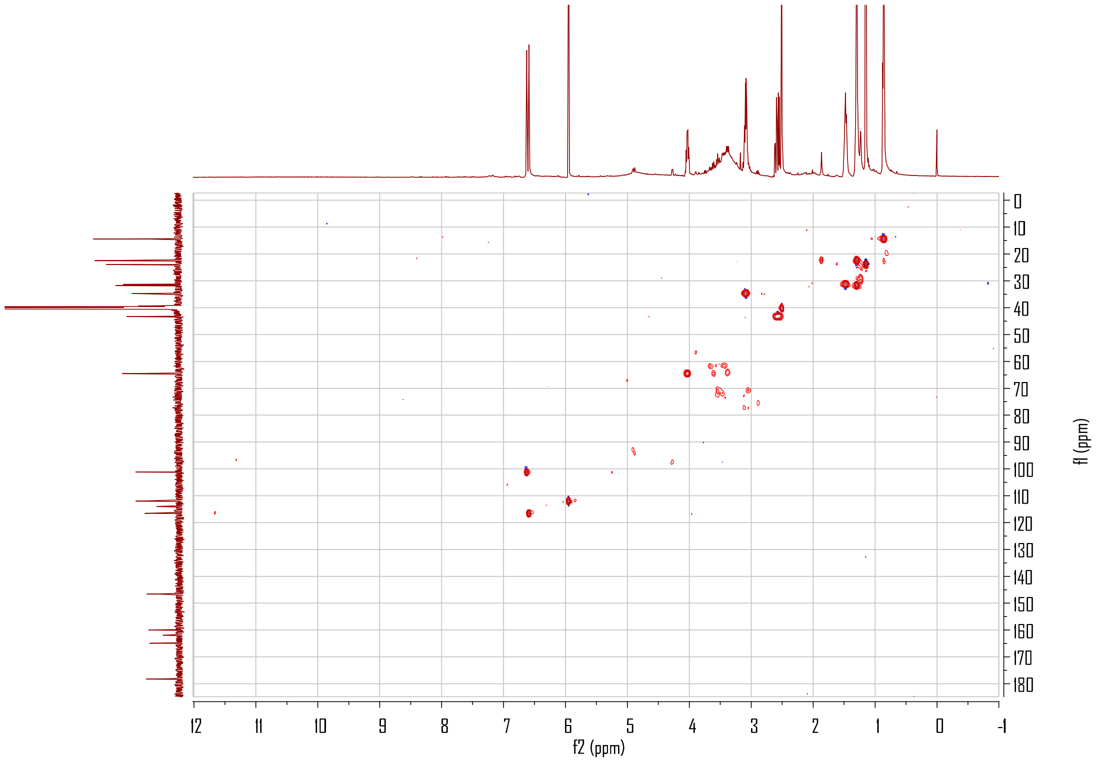

Figure S20. HSQC spectrum of compound **4** (500 MHz, DMSO-*d*6).

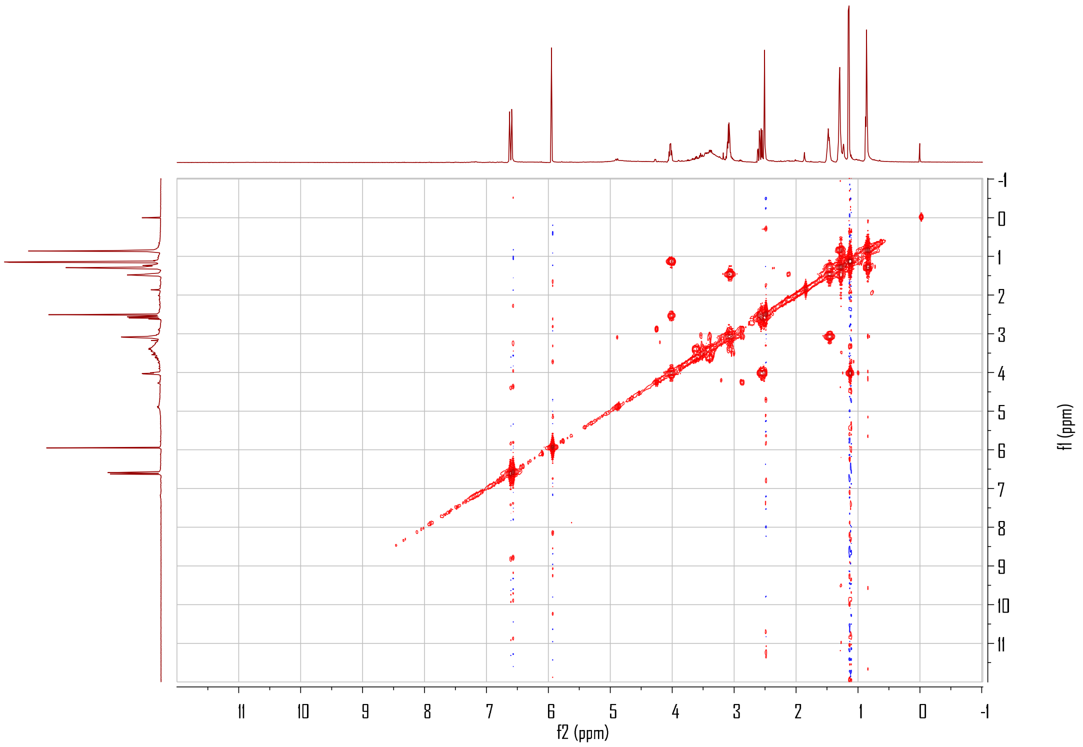

Figure S21. COSY spectrum of compound **4** (500 MHz, DMSO-d_6).

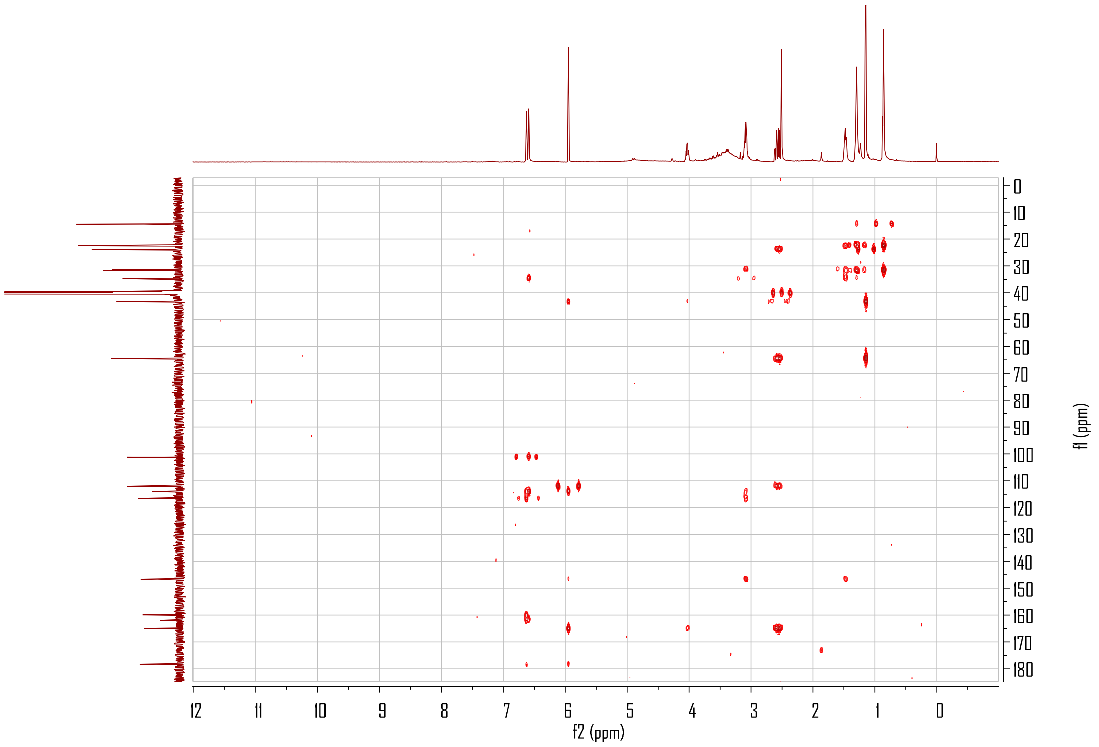

Figure S22. HMBC spectrum of compound **4** (500 MHz, DMSO-d_6).

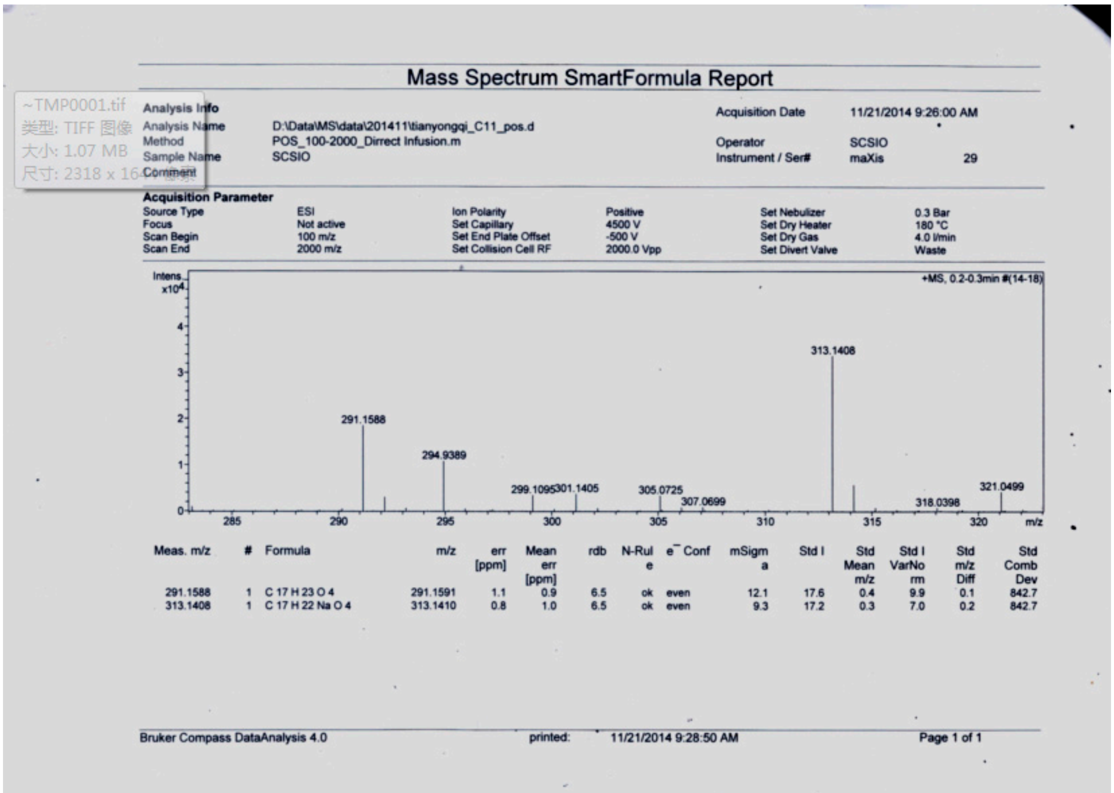

Mass Spectrum SmartFormula Report

~TMP0001.tif
类型: TIFF 图像
大小: 1.07 MB
尺寸: 2318 x 16

Analysis Info

Analysis Name	D:\Data\MS\data\201411\tianyongqi_C11_pos.d	Acquisition Date	11/21/2014 9:26:00 AM
Method	POS_100-2000_Dirrect Infusion.m	Operator	SCSIO
Sample Name	SCSIO	Instrument / Ser#	maXis 29
Comment			

Acquisition Parameter

Source Type	ESI	Ion Polarity	Positive	Set Nebulizer	0.3 Bar
Focus	Not active	Set Capillary	4500 V	Set Dry Heater	180 °C
Scan Begin	100 m/z	Set End Plate Offset	-500 V	Set Dry Gas	4.0 l/min
Scan End	2000 m/z	Set Collision Cell RF	2000.0 Vpp	Set Divert Valve	Waste

Meas. m/z	#	Formula	m/z	err [ppm]	Mean err [ppm]	rdb	N-Rule	e⁻ Conf	mSigma	Std I	Std Mean m/z	Std I VarNorm	Std m/z Diff	Std Comb Dev
291.1588	1	C 17 H 23 O 4	291.1591	1.1	0.9	6.5	ok	even	12.1	17.6	0.4	9.9	0.1	842.7
313.1408	1	C 17 H 22 Na O 4	313.1410	0.8	1.0	6.5	ok	even	9.3	17.2	0.3	7.0	0.2	842.7

Bruker Compass DataAnalysis 4.0 printed: 11/21/2014 9:28:50 AM Page 1 of 1

Figure S23. HRESIMS spectrum of compound **4**.
